# Transanal Resection of a Prolapsed Rectal Lipoma: A Report of a Rare Case

**DOI:** 10.7759/cureus.78247

**Published:** 2025-01-30

**Authors:** Nahlah S Arab, Rawan K Alkhatabi, Abdullah F Alhalafi, Mohammed B Beyari, Raghad A Alahmadi, Abdulah A Almazro

**Affiliations:** 1 Department of General Surgery, Consultant in Colorectal Surgery, Prince Sultan Military Medical City, Riyadh, SAU; 2 Saudi Board General Surgery Residency Program, King Fahad General Hospital, Jeddah, SAU; 3 Saudi Board General Surgery Residency Program, Prince Sultan Military Medical City, Riyadh, SAU; 4 ‎‏College of Medicine, King Saud University, Riyadh, SAU; 5 College of Medicine, Taibah University, Madinah, SAU; 6 College of Medicine, King Saud ‎‏University, Riyadh, SAU

**Keywords:** abdominal mri, colonoscopy, rectal lipoma, surgical resection, transanal resection

## Abstract

Lipoma is a benign tumor that arises from mesenchymal cells and is considered relatively rare. Although lipomas can develop anywhere in the digestive tract, they are seldom found within the intestinal tract. Typically asymptomatic, colonic lipomas usually do not require treatment unless they result in symptoms that warrant surgical intervention.

In this case, a 68-year-old male patient presented to the colorectal clinic with complaints of chronic constipation lasting five years, along with a bulging protrusion through the anus that could be reduced by the patient's finger. He reported no history of abdominal pain, change in bowel habits, rectal bleeding, melena, weight loss, fever, or night sweats. During colonoscopy, large subepithelial lesions, measuring greater than 2 cm, were observed proximal to the anal verge. This case report highlights the significance of accurately diagnosing colonic lipomas to prevent potential misdiagnoses as malignancies or rectal prolapses. Additionally, we advocate for treatment strategies tailored to several factors, including the lipoma's size, location, and any associated symptoms.

## Introduction

Bauer described the first case of gastrointestinal lipomas in 1757. Even though they are rare, they have been reported internationally over the last few decades and centuries [1,2]. Gastrointestinal lipomas can be classified into three distinct pathological types: submucosal, intermuscular, and subserosal. The literature indicates that submucosal lipomas are the most common [3]. Gastrointestinal lipomas are primarily found in the colon, with colonic lipomas typically originating in the ascending colon, followed by the transverse colon, descending colon, sigmoid colon, and rectum [4]. Research on rectal lipomas is limited, with cases reported in relatively small numbers [5]. Colonic lipomas are usually asymptomatic and do not require treatment unless they exceed 2 cm in diameter, which may cause symptoms necessitating surgery [1,6]. There can be a wide variety of symptoms based on the tumor's location and size, potentially involving abdominal pain, obstructions, perforations, intussusceptions, changes in bowel habits, rectal bleeding, prolapses, and even massive hemorrhages. In giant lesions, superficial ulceration may occur, resulting in hemorrhage and various symptoms [7,8]. One of the most significant clinical implications of colonic lipoma is its potential to be misdiagnosed as colonic malignancy, due to its symptomatic similarities [9]. Differentiating between lipomas and carcinoma is essential, as the two conditions require vastly different surgical approaches. Carcinoma is often associated with loss of appetite and unintentional weight loss. Lipomas are generally found on the right side, while epithelial neoplasms tend to occur on the left [8].

Although tissue excision is required to establish the ultimate diagnosis, imaging techniques aid in the diagnosis. A smooth, oval filling defect can be observed using barium X-rays, while endoscopic ultrasonography shows that non-vascular, hyperechoic submucosal lesions may supplement the diagnosis. Colonic lipomas >2 cm can be detected using computed tomography (CT), by showing sharp margins in homogeneous fat density, and magnetic resonance imaging (MRI), by distinguishing lesions in adipose tissue density [8].

In the case of asymptomatic colonic lipomas, the treatment entails observation, while surgical intervention is based on the size, location, and presence or absence of complications; surgeons decide whether to perform endoscopic or surgical intervention. Surgical intervention is necessary for conditions such as obstruction, intussusception, perforation, or massive hemorrhages. Evidence shows that endoscopic removal of submucosal lipomas up to 2 cm in diameter is safe, effective, and appropriate. Laparotomies with enucleation, colotomies, excisions, and segmental colonic resection have all been described as choices of treatment, as have minilaparotomies and transanal resections. A selected number of patients may also be candidates for minimally invasive surgeries, such as laparoscopic-assisted resection under colonoscopic guidance [8].

Until now, no scientific studies have determined the most reliable methods for detecting and managing rectal lipomas. Consequently, most patients receive treatment based on case reports in the literature. This report presents a rare case of rectal lipoma, intending to highlight its clinical significance and discuss the various approaches to diagnosis and management.

## Case presentation

**Figure 1:**
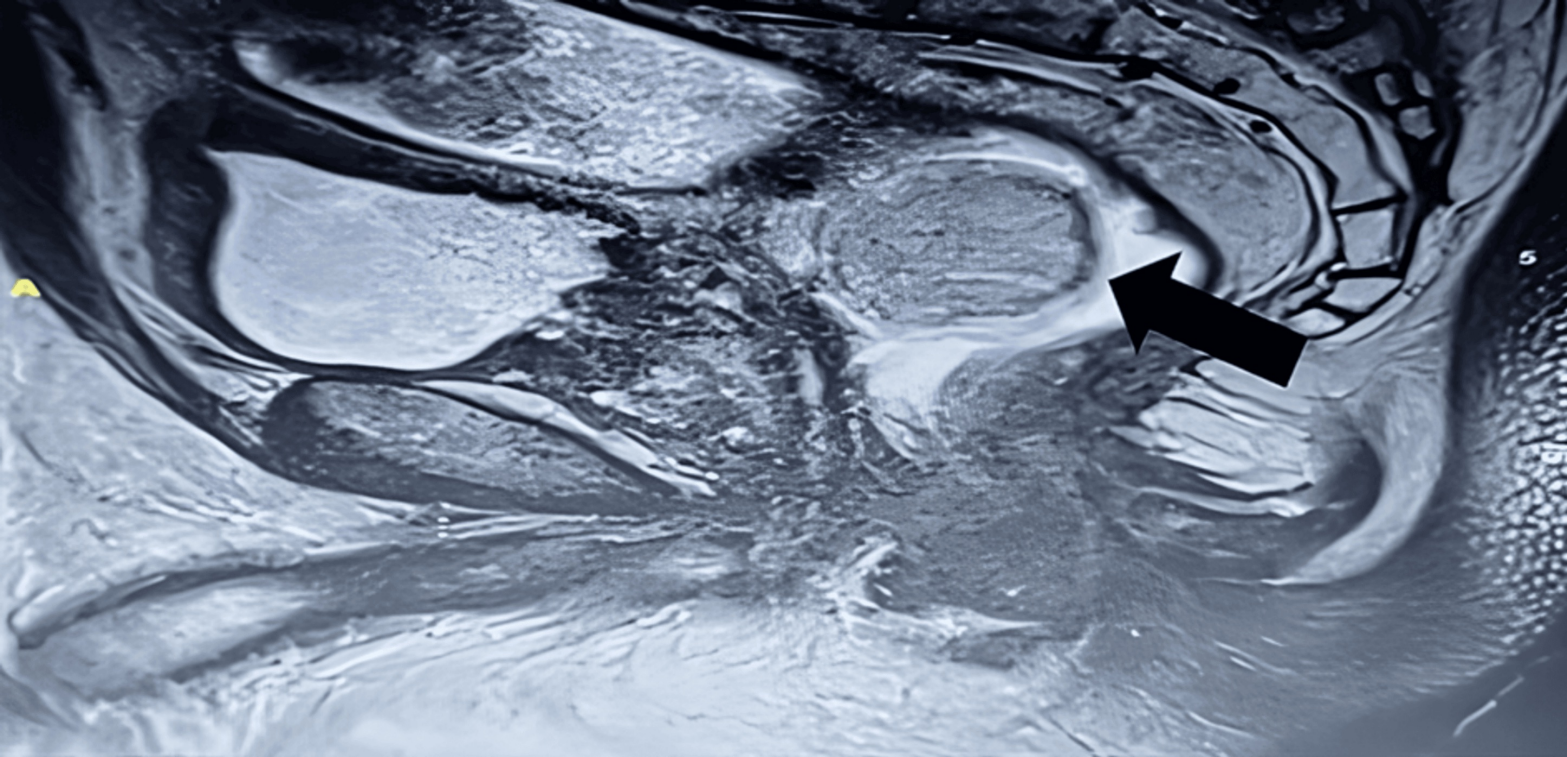
An MRI image shows that the tumor was located in the mid-rectal mass, 7.6 cm from the anal verge, measuring 3.5 x 4 x 3.4 cm, with intermediate T2 and high T1 signal intensity (arrow). MRI, magnetic resonance imaging

A 68-year-old male patient, surgically free, with a history of diabetes and hypertension, presented to the colorectal clinic complaining of chronic constipation for five years, associated with a bulging protrusion through the anus, which could be reduced by the patient's finger. There is no history of abdominal pain, changes in bowel habits, rectal bleeding, melena, weight loss, fever, or night sweats. There is no family history of similar complaints or malignancies. Upon examination, the patient appeared to be in good health, conscious, alert, oriented, vitally stable, afebrile, and saturating well in room air. During the local examination, the abdomen was soft and lax, neither distended nor tender; the per rectum (PR) exam revealed a doughy, mobile, well-circumscribed, non-tender, and hyperemic mass. No skin tags, anal fissures, or hemorrhoidal tissue were noted. Colonoscopy was advanced up to the sigmoid colon but could not be advanced further due to poor bowel preparation, and there was a large subepithelial lesion of more than 2 cm just proximal to the anal verge. In MRI defecography, an incidental left polypoid mid-rectal mass was located 7.6 cm from the anal verge, measuring 3.5 x 4 x 3.4 cm, showing intermediate T2, high T1 signal intensity, a complete signal drop on fat-saturation images, and no restricted diffusion, which indicated lipoma characteristics (Figure 1).

**Figure 2:**
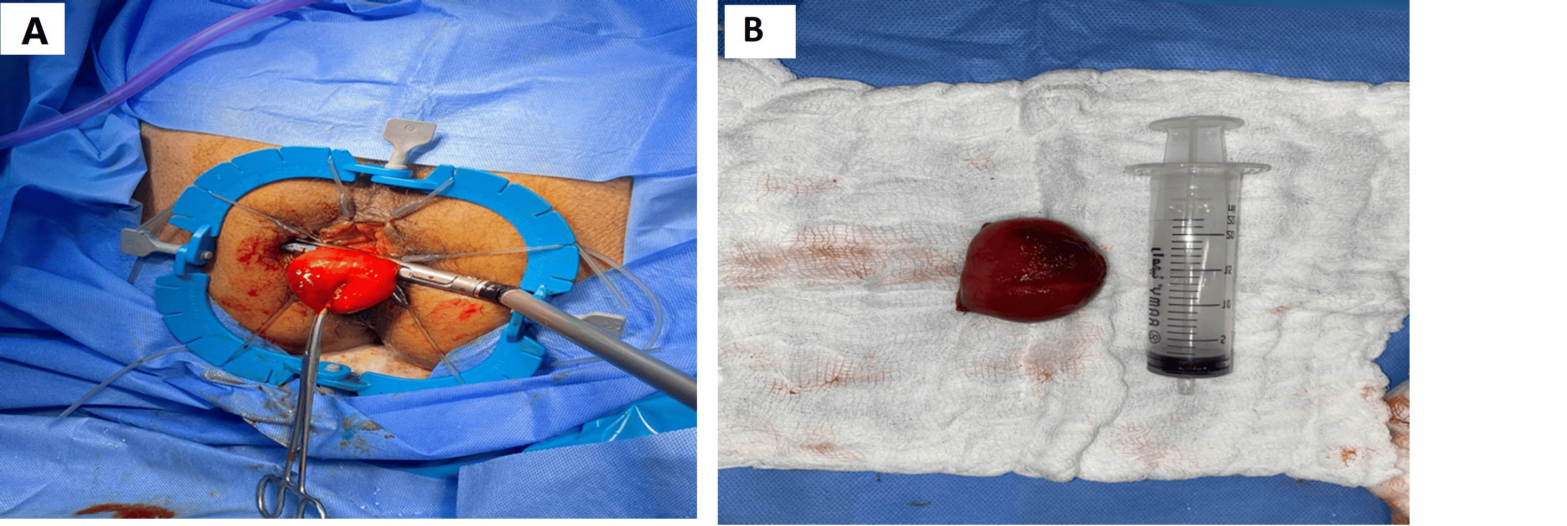
(A) A huge lipoma that extends through the anus; (B) Gross view of the resected rectal lipoma.

Based on clinical information, the colonoscopy report, and the MRI results, we decided to admit the patient and proceed with transanal rectal mass excision. Intraoperatively, we discovered a pedunculated rectal mass spontaneously protruding beyond the anal verge at 12-3 o'clock (Figure 2A). In addition to being soft and yellow, the mass had a smooth surface and a fine texture (Figure 2B). We sent the mass to histopathology, which showed mature fibroadipose tissue, consistent with lipoma. A smooth postoperative course was observed, and the patient is doing well, passing a bowel motion. On his first postoperative day, he was discharged from our hospital. He followed up in the clinic, where he was doing well.

## Discussion

Lipoma is a benign and rare tumor originating from mesenchymal cells. After adenomatous polyps, lipomas are considered the second most common benign tumors of the colon [10-14]. According to the data, the incidence of this disease has ranged from 0.035% to 4.4% [12,13]. In most cases, it affects individuals between the ages of 50 and 65 [7,8,11,12]. However, younger patients can also develop these lesions [15]. Some studies report that females predominate among cases by as much as 66.7% [7], while other studies report that the condition is equally prevalent among males and females [8]. Interestingly, women are more likely to develop colonic lipomas in the right colon, while men are more likely to develop them in the left [16].

Intestinal lipomas can be classified into three pathological types: submucosal, intermuscular, and subserosal. Submucosal lipomas develop from the submucosal layer and project into the lumen. They are the most common and make up over 90% of intestinal lipomas. Intermuscular lipomas are found within the muscular layers. Subserosal lipomas arise from the serosa and protrude out of the gastrointestinal tract [3]. The size of intestinal lipomas ranges from 2 mm up to 30 cm [16]. They are usually solitary, but there may be multiple cases in 6%-20% of cases [17].

It has been suggested that certain factors may contribute to this condition, including chronic irritations, inflammations, and excessive fat accumulation due to an underdeveloped arterial, venous, and lymphatic system [12]. However, no links have been found with diabetes, hyperlipidemia, or other degenerative diseases [1].

Lipomas are well-differentiated tumors that develop from adipose connective tissue [16]. They are characterized by a smooth, rounded mass, either pedunculated or sessile, with a yellowish color [4,7,8,12,14]. Microscopically, they appear as lipohyperplasia or a diffuse increase in adipose tissue in the submucosa [1].

A majority of events occur in the ascending colon (61%) [7,11,18]. Approximately 20.1% of these lesions are found in the descending colon, 15.4% in the transverse colon, and 3.4% in the rectum [7,12,19,20]. Despite the variability, more than 94% of these lesions remain asymptomatic and are detected during routine endoscopic procedures [3,18,21].

One of the most common symptoms of colonic lipomas is mild rectal bleeding. Lipomas on the right side of the colon present as palpable masses, while those on the left are associated with obstructive symptoms. However, clinical manifestations are more closely related to the lipoma size than to the colon segment affected [17].

A lipoma of more than 2 cm is typically associated with abdominal pain (42.4%), rectal bleeding (54.5%), and changes in bowel habits (24.2%) [4,12,14,22]. In addition, constipation, intussusception, hemorrhage, obstruction, and anemia may also result from this condition [4,18,23,24].

One of the primary challenges in managing colonic lipomas is their tendency to mimic some diseases, resulting in significant diagnostic uncertainty [25]. Common misdiagnoses include rectal prolapse and malignancy [12]. In colonic lipomas, no sarcomatous changes have been reported, but frequent torsion and relative ischemia can cause a pseudomalignant appearance. A malignant colonic neoplasm and a colonic lipoma typically occur in similar age groups and show comparable symptoms. The preoperative diagnosis holds clinical significance [16].

Barium enema studies can provide initial insights by revealing smooth, radiolucent septate intraluminal filling defects that elongate during peristalsis, also known as the "squeeze sign" [4,7,13,23]. However, this imaging modality lacks the specificity necessary to differentiate lipomas from other neoplastic masses effectively [13].

Endoscopic ultrasonography is regarded as a highly beneficial diagnostic tool. It effectively identifies lipomas as hyperechoic lesions and can assess their depth within the colonic wall [8,12-14]. The depth measurement is crucial in determining whether the lesion can be removed via endoscopic resection.

Additionally, colonoscopic examinations have been linked to three distinctive signs of colonic lipomas that assist in diagnosis: the "tent sign" (lifting of the mucosa over the lipoma using forceps), the "cushion sign" (indentation of the lipoma with forceps that resolves upon release), and the "naked fat sign" (extrusion of adipose tissue during biopsy) [7,12,23].

When patients experience severe illness or present with large lipomas, CT and MRI are valuable diagnostic tools due to their capacity to characterize and identify the typical imaging features of adipose tissue [11,19]. However, the effectiveness of CT may be compromised in cases involving very small lesions or the partial volume effect, which can result in the failure to detect smaller lesions and potentially lead to an overestimation of larger masses due to the presence of adjacent fecal matter or soft tissue [12,24].

Although recent innovations in radiology diagnostics have emerged, histopathologic evaluation remains the gold standard for accurate diagnosis [9]. Reports show that the preoperative diagnostic accuracy is only about 62% [16].

The optimal management of colonic lipomas depends on their size, symptoms, and any associated complications. Generally, asymptomatic lipomas are observed [25], while symptomatic or complicated ones necessitate intervention. For lesions smaller than 2 cm, endoscopic excision using snares and electrocautery is preferred, as larger lipomas carry a heightened risk of perforation during endoscopic removal [8,10,26]. Recent advancements in endoscopic techniques, including submucosal resection, have facilitated the excision of larger lesions [2,27].

Segmental colectomy, along with lipectomy, remains the gold standard for managing lipomas larger than 2 cm or those complicated by significant issues [12,28]. Hemicolectomy is typically reserved for very large, wide-based subserosal lesions that cause excessive bleeding or intussusception [14]. Laparoscopic removal is a less invasive option compared to endoscopic methods when they are either less achievable or safe [17,29]. The advantages of laparoscopic procedures include reduced postoperative pain and a quicker recovery time [14,16,17,21,30].

In our case, the rectal lipoma presented as a polypoidal lesion that the patient manually repositioned through the anus. This was confirmed by MRI and colonoscopy, leading to an accurate preoperative diagnosis that helped prevent unnecessary delays and potential misdiagnoses, such as malignancy or rectal prolapse. The successful transanal excision surgery underscores the importance of combining imaging findings with clinical presentation in effectively managing symptomatic rectal lipomas. This case highlights the advantages of a minimally invasive approach, as the patient experienced a smooth postoperative recovery and was discharged the following day without complications.

## Conclusions

In conclusion, colorectal lipoma is a rare condition predominantly observed in elderly individuals. The symptoms can vary significantly depending on the location and size of the tumor, potentially leading to abdominal pain, obstructions, perforations, intussusceptions, alterations in bowel habits, rectal bleeding, prolapses, and even severe hemorrhages. A range of diagnostic modalities, including colonoscopy and CT, are available to aid in diagnosis; however, histopathologic evaluation remains the gold standard for accurate diagnosis. This case report emphasizes the importance of precise diagnosis and appropriate management of colonic lipomas, especially in atypical locations such as the rectum. It highlights the value of integrating clinical presentation with advanced imaging techniques, such as MRI and colonoscopy, to achieve accurate preoperative diagnoses and avoid potential misdiagnosis as malignancy or rectal prolapse. Furthermore, it underscores the need for tailored treatment strategies that take into account various factors, including the size and location of the lipoma, as well as any associated symptoms, reinforcing the importance of individualized patient care in colorectal surgery. The successful transanal excision of the symptomatic rectal lipoma illustrates the effectiveness of minimally invasive techniques, resulting in a smooth postoperative recovery for the patient.
